# Tricyclic Antidepressant and Antipsychotic Toxicity: Clomipramine and Ziprasidone Overdose

**DOI:** 10.7759/cureus.63691

**Published:** 2024-07-02

**Authors:** Nikolas P Foresteire, Cory Howard, Kirk Szustkiewicz

**Affiliations:** 1 Emergency Medicine, Hospital Corporation of America (HCA) Brandon Hospital, Brandon, USA; 2 Emergency Medicine, Morsani College of Medicine, University of South Florida, Graduate Medical Education Consortium, Tampa, USA

**Keywords:** atypical antipsychotic, toxicology and poisoning, toxicology, medical toxicology, ziprasidone, tricyclic antidepressant overdose, tricyclic antidepressant, tca toxicity

## Abstract

This case report details an intentional overdose attempt utilizing tricyclic antidepressants (TCAs) and atypical antipsychotics with significant neurologic, pulmonary, and cardiac toxicity. In conjunction with the local poison control center, progression of the clinical toxidrome was anticipated, aggressively managed, and successfully treated. This case highlights the dangers of significant TCA toxicity, peak onset of toxicity within six hours, and the amplification of clinical toxidromes with co-ingestions.

## Introduction

Tricyclic antidepressants (TCAs) were approved for distribution in the United States (US) during the 1950s [1]. Initially designed for the treatment of melancholic depression, imipramine was the first TCA of its kind. After the initial development, additional classes and formularies of TCAs became available. Although they may be utilized for treating many clinical syndromes, their frequent dose adjustments, slow titration to effect, and narrow therapeutic windows prompted the invention of serotonin reuptake inhibitors in the 1980s [1]. In addition, the side-effect profile for therapeutic doses includes confusion, orthostatic hypotension, cardiac dysrhythmias, constipation, lowered seizure threshold, and serotonin syndrome in some individuals [2]. As a result, TCAs have seen a large decline in prescription volume but remain a second-line therapy for depression. A vast list of on and off-label uses for the treatment of various conditions including insomnia, attention deficit hyperactivity disorder, panic attacks, phobias, anxiety, migraine prophylaxis, and chronic pain syndromes still exists today [2].

In the US, TCAs accounted for 4705 single toxic exposures, resulting in 15 deaths reported by the *2022 Poison Control Centers’ National Poison Data System Annual Report *[3]. In acute TCA overdoses, cardiac dysrhythmias and hypotension are strong predictors of mortality. Typically, ingestions greater than 1 gram/kg are fatal [4]. Multiple generations of atypical antipsychotics exist, with proven benefits for anxiety, obsessive-compulsive disorder, mood stabilization, and schizophrenic symptoms. Atypical antipsychotics also carry a high risk of cardiac dysrhythmias, orthostatic hypotension, extrapyramidal symptoms, sedation, delirium, and dystonia. As a result of simultaneous co-ingestions and high risk for clinical decompensation, a clinical conundrum of mixed toxidromes for emergency medicine physicians can be created. Atypical antipsychotics and TCA co-ingestions may amplify the degree of clinical toxicity. Immediate stabilization and timely management of acute toxicity are vital. In this case report, we aim to identify and outline the TCA toxidrome in conjunction with co-ingestion of atypical antipsychotics.

## Case presentation

The patient was a 20-year-old female, with a prior medical history of depression, schizophrenia, and multiple previous suicide attempts, who presented to the emergency department via emergency medical services (EMS) after being found unresponsive. On scene, EMS noted that the patient had admitted to her mother “that she wanted to die” before becoming progressively more unresponsive. She was found to have access to 50 mg Ziprasidone and 40 mg Clomipramine with unknown pill counts. She was minimally responsive with pinpoint pupils bilaterally. Point of care glucose was 30 mg/dL and the patient was not protecting her airway. On route to the emergency department, she was bradypneic and tachycardic. Respiratory support was provided by EMS (ALS crew) with 4 liters of supplemental oxygen via a nasal cannula, right antecubital IV access was established, and she was given a total of 50 cc of 10% dextrose. A repeat point-of-care glucose was drawn upon arrival and found to be 60 mg/dL. On arrival, her body temperature was 36.4°C (oral), heart rate 121 beats/minute, respiratory rate 9 breaths/minute; and blood pressure 139/77 mmHg. Our physical examination revealed that the patient was somnolent, pale/clammy appearing, and unresponsive to sternal rubbing. She had shallow respirations with coarse breath sounds bilaterally. Her pupils were pinpoint bilaterally and minimally responsive to light. Although mydriasis may be common in acute TCA overdose, it is suspected the patient's miosis was likely a result of alpha-1 receptor blockade. She also had delayed capillary refill in all extremities and a Glasgow Coma Scale Score of 3. 

Emergent interventions

Rapid sequence Intubation was performed using 36 mg IV rocuronium and 50 mcg IV fentanyl. The patient was intubated without difficulty due to our concern for airway protection and she was placed on 10 mg/mL titrated propofol drip. A nasogastric tube was inserted. She was given 1 liter of 0.9% sodium chloride, 50 mL dextrose 50%, and 500 mL dextrose 10% due to hypoglycemia (49 mg/dL). IV piperacillin/tazobactam was administered (3.375 g) due to concern for aspirational pneumonia.

Findings

**Figure 1:**
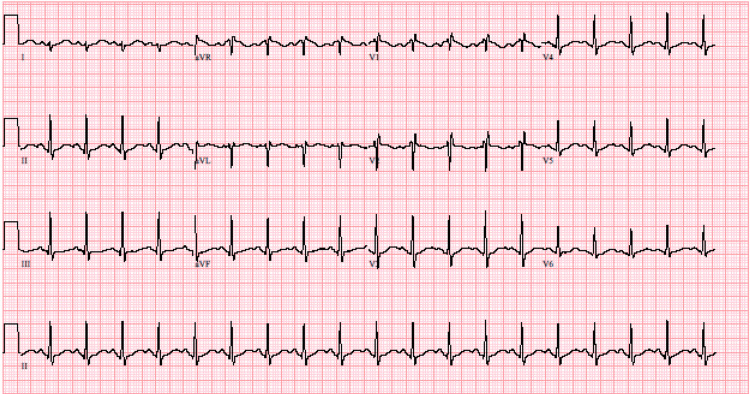
Electrocardiogram of Patient X.

Electrocardiogram (ECG) (Figure 1) was notable for sinus tachycardia at a rate of 115 beats/min, an incomplete right bundle branch block, and non-specific ST/T-wave changes. The QRS duration was 96 milliseconds. QT/QTc intervals were noted to be prolonged at 370/511 milliseconds. The patient's lactic acid was elevated (4.5 mmol/L) with screens for salicylates, acetaminophen, and ethyl alcohol negative, but a urine drug screen for THC was presumptive positive. 

**Figure 2:**
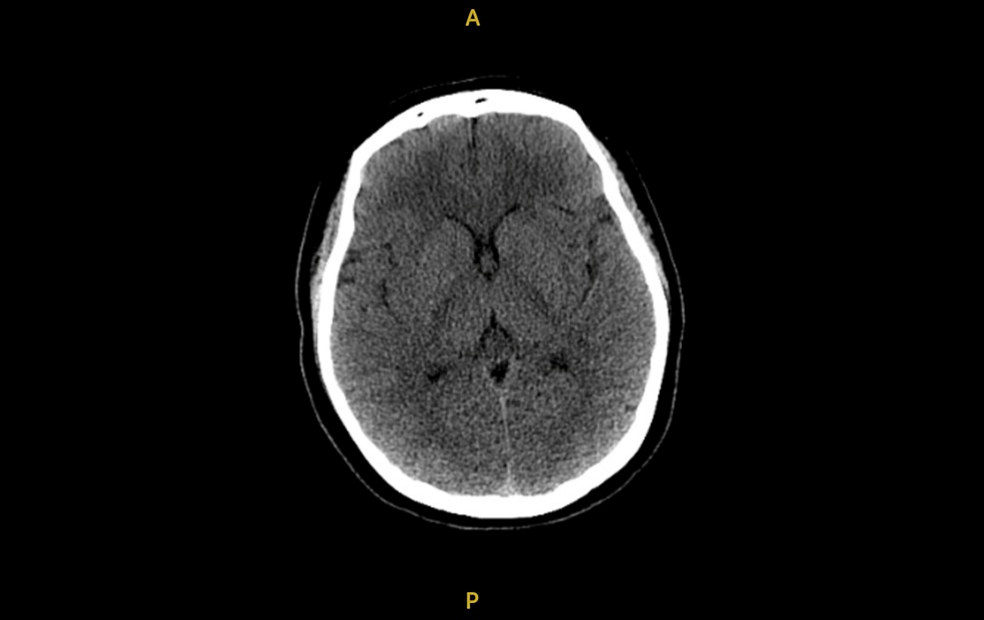
Non-contrast computed tomography (CT) brain of Patient X.

**Figure 3:**
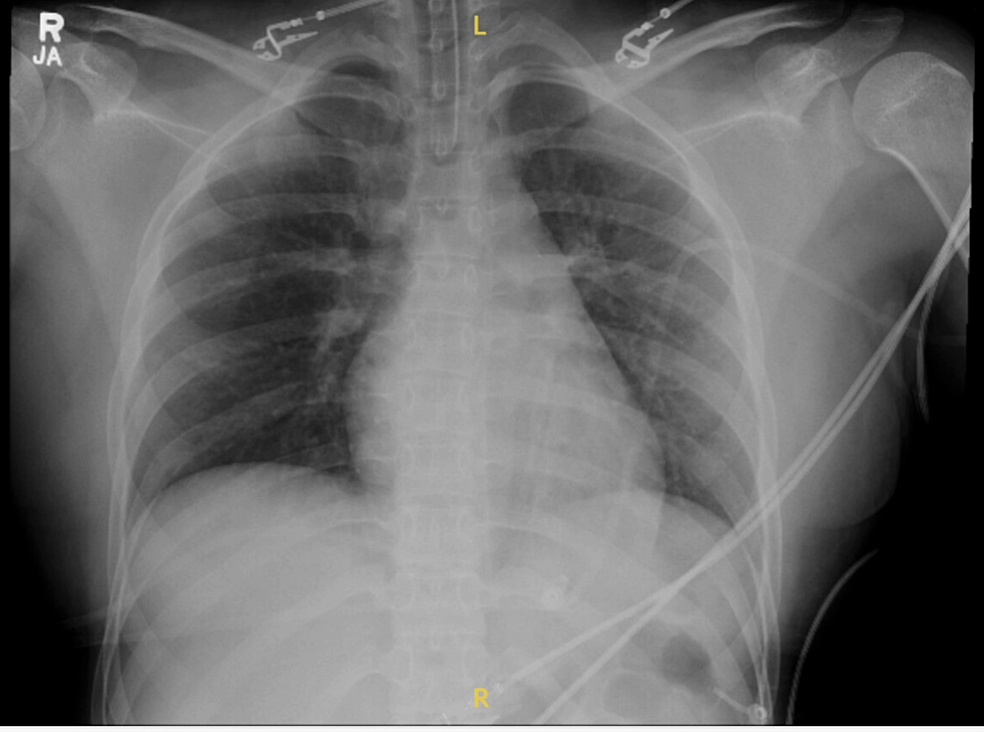
Chest radiography (Chest X-ray) of Patient X.

A CT brain without contrast (Figure 2) was performed and was notable for mild cerebral edema with concern for possible anoxic brain injury. The chest X-ray (Figure 3) obtained after intubation was unremarkable. 

The local poison control center was contacted and made recommendations for 2 grams IV magnesium sulfate and optimizing potassium greater than 4 mmol/L. Additional recommendations included continued monitoring for prolonged QTC, QRS duration greater than 100 ms, and evaluation for possible seizures or neuroleptic malignant syndrome. The patient was admitted to the intensive care unit in critical condition with the diagnosis of a suicide attempt with suspected TCA and ziprasidone overdose.

On day 3 after admission, the patient was successfully extubated. The patient confirmed that her ingestion was intentional. Total pill counts were confirmed including 29 ingested tablets of 50 mg Clomipramine and 55 ingested tablets of 40 mg Geodon. Both prescriptions were filled eight days before clinical presentation. The patient noted that she was non-compliant with the administration of medications at prescribed intervals and often missed her scheduled dosing before intentionally overdosing. She was neurologically intact, oriented back to baseline, and was subsequently discharged to an inpatient psychiatric treatment facility five days later.

This case highlights the dangers of TCA toxicity in suicidal attempts and amplification via co-ingestion with atypical antipsychotics resulting in the need for multimodal treatment and close monitoring.

## Discussion

We present the case of a young woman who intentionally overdosed utilizing TCAs and other atypical antipsychotics and responded well to intubation, fluid resuscitation, and electrolyte optimization. While we do not have an exact pill count, our case highlights the associated dangers of these often-prescribed medications and the predicted toxidrome. 

Tricyclic antidepressants

There are eight formularies of TCAs readily available in the US. TCAs primarily exert pharmacologic effects by inhibiting the reuptake of norepinephrine and serotonin in the presynaptic cleft [1]. These drugs also exhibit antagonism of central and peripheral muscarinic acetylcholine receptors, peripheral alpha-1 adrenergic receptors, histamine-1 receptors, CNS gamma-aminobutyric acid A receptors, and blockade of cardiac fast sodium channels. They are highly lipophilic, protein and plasma-bound, and readily cross the blood-brain barrier. Peak onset is within 2-6 hours of ingestion [1]. Depending on the TCA, the half-life may be 24-60 hours [4]. TCAs are eliminated from the body utilizing phase-one hepatic metabolism via CYP2D6, CYP2C19, and phase-two glucuronidation [5]. Most TCAs have active metabolites, which increases the time of observed clinical toxicity. There are two classifications of TCAs as outlined in Table 1. Also, TCA mechanism(s) of action and clinical effects are presented in Table 2.

**Table 1:** Tricyclic antidepressants: amine classifications, generic drug names, brief mechanism of action. TCA: Tricyclic antidepressant

TCA Class	Drugs	Notes/General Mechanism
Secondary Amines (1 methyl side-chain)	Desipramine (most potent and adrenergic) [Higher fatality rate], Nortriptyline (most commonly used for geriatric depression), Protriptyline (less commonly prescribed)	More potent norepinephrine reuptake inhibitor > serotonin reuptake inhibitor Tolerated with fewer side-effects/”dirty receptor interactions”
Tertiary Amines (2 methyl side-chains)	Amitryptyline, Clomipramine (most serotonergic), Doxepin (most histaminergic), Imipramine	More potent serotonin reuptake inhibitor > Norepinephrine

**Table 2:** Tricyclic antidepressant mechanism(s) of action and clinical effects.

Mechanism	Clinical Effect(s)	Toxidrome(s)
1. 5-HT, NE, DA reuptake inhibitor	Antidepressant effects	Sympathomimetics, myoclonus/ hyperreflexia, serotonin syndrome
2. Anticholinergic (M1 blockade)	Sedation + Tachycardia	Central (agitation, delirium, confusion, hallucinations, slurred speech, ataxia, seizures, sedation, coma) or peripheral (mydriasis, ileus, urinary retention, tachycardia)
3. Na+ channel blockade	QRS prolongation	Negative inotropy, hypotension, bradycardia, heart blocks, ectopy
4. Alpha-1 inhibitor	Vasodilation + Hypotension	Sedation, refractory hypotension (MCCOM), miosis
5. Histamine blockade (H1)	Sedation + Seizures	Coma
6. GABA-A antagonism	Seizure contribution	Seizures
7. Potassium-channel inhibition	QT prolongation + Ectopy	Ventricular dysrhythmias

Serious toxicity from TCAs most commonly occurs within six hours of ingestion. As a general rule, ingestions less than 1 mg/kg are typically non-toxic. Life-threatening ingestions may range between 10 mg/kg and 20 mg/kg with severe central nervous system and cardiovascular toxicity. Ingestions greater than 1 gram/kg are typically fatal [4]. While the precise amount of clomipramine ingested by our patient is unknown, we can predict that she likely ingested less than 20 mg/kg given her clinical appearance and outcome, which may have proven fatal if not addressed.

Clinical toxidromes

Cardiotoxicity carries the highest mortality risk of all clinical findings. Our patient experienced tachycardia and incomplete bundle branch block along with mild QTc prolongation. We optimized her potassium, magnesium, and calcium, thus shortening her QTc interval and decreasing her risk for cardiac dysrhythmias. Cardiotoxic effects occur via the inhibition of fast sodium channels in the His-Purkinje System and myocardium, resulting in decreased conduction velocity, leading to an increased rate of polarization, which prolongs the absolute refractory myocardial period. TCAs also block myocardial potassium channels and the efflux of potassium during repolarization. This may manifest as QT interval prolongation, bradycardia, ectopy, heart blocks, Brugada pattern, Torsades de pointes, and rapidly degenerate into ventricular arrhythmias [4]. 

Most ECG abnormalities arise within six hours and resolve after 36-48 hours. The most common finding will be sinus tachycardia. Interval changes are of higher clinical concern during suspected TCA toxicity. Our patient had sinus tachycardia and QT interval changes within six hours of presentation; however, her QRS interval remained less than 100 ms. There is a 34% increased risk for seizure when a QRS is greater than 100 ms and a 50% increased risk for cardiac dysrhythmias when a QRS has prolonged greater than 160 ms [6]. If there is rate-dependent QRS prolongation, this is indicative of significant sodium channel toxicity. Additionally, non-specific ST segment elevations or depression, T-wave changes, right axis deviation, terminal R-wave in lead aVR (> 3 mm), and S-waves in leads I and aVL may be present. If absent, the lack of these ECG findings carries a 94% negative predictive value for TCA toxicity [7].

Clinical evaluation

When working up a patient with an undifferentiated toxidrome, it is prudent to obtain laboratory results including acetaminophen, salicylates, UDS, and alcohol. Almost half of all TCA overdoses involve a co-ingestion. Of note, UDS may show false TCA positive due to side chain recognition from common medications including cyclobenzaprine, carbamazepine, cetirizine, cyproheptadine, diphenhydramine, hydroxyzine, quetiapine, and phenothiazines.

Clinical management

Gastrointestinal (GI) decontamination may be effective in patients with TCA toxicity. Gastric lavage has been shown to have limited efficacy but may be beneficial if utilized within 1-2 hours of a large dose ingestion. It is recommended to consult a local poison control center for recommendations before performing GI decontamination. Removing large quantities of pills with lavage may significantly reduce the likelihood of fatal systemic toxicity. 

Activated charcoal

Given the unknown time of ingestion, altered mentation, and high risk for aspiration, activated charcoal was not recommended for our patient. In the setting of suspected ingestion, data supports the recommended use of single-dose activated charcoal within one hour post-ingestion. Initial charcoal dosing is 1 gram/kg by mouth in alert, oriented, and PO-tolerant adult patients. Do not administer this medication to those with contradictions including vomiting, altered mental status, or those clinically deemed to be at high risk of aspiration. If aspirated, this may cause pulmonary obliterans [4]. However, in those with a secured airway, activated charcoal may be considered to reduce the progression to systemic absorption, distribution, and potentially fatal toxicity.

Sodium bicarbonate

Sodium bicarbonate is indicated for a QRS duration greater than 100-110 ms or hypotension that is refractory to IV fluid administration [1,4]. Initial bolus dosing is 1-2 mEq/kg [1]. It is recommended that you repeat an electrocardiogram three to five minutes after bolusing to evaluate for response to dosing with QRS narrowing [8]. After administration of bolus dosing, an infusion should be started. Institutional policy should be followed. Sodium bicarbonate (3 amps) may be placed in 1L of D5W and started at double maintenance rate dosing (2-3 mL/kg/hr). With sodium bicarbonate, pH must be closely monitored and should not be allowed to exceed 7.55. Additionally, potassium must be closely monitored as hypokalemia is a common finding.

Seizures

Seizures should be treated aggressively with benzodiazepines. Preferred agents include diazepam and lorazepam. If status epilepticus refractory to benzodiazepine administration develops, phenobarbital may be utilized at 10-15 mg/kg bolus dosing [4]. Rapid sequence intubation and a definitive airway should be established. After intubation, versed and propofol drips may be used as indicated, due to their anti-epileptic properties. Further seizure workup including a STAT bedside electroencephalogram must be performed in conjunction with a neurologist to ensure cessation of epileptic brain activity. Monitoring for rhabdomyolysis with repeat serum creatinine kinase is also reasonable in patients with prolonged seizure activity. Further pharmaceutical management should be followed as indicated (i.e.: levetiracetam).

Refractory hypotension

If hypotension is present, IV fluids should be administered in a 30 cc/kg bolus [4]. If refractory hypotension is present, levophed or/and epinephrine infusions should be started and titrated as needed. Levophed and epinephrine have been shown to compete with TCAs directly at the alpha-adrenergic receptors [4,9]. Recommended dosing includes 1 microgram/minute and titrating to a mean arterial pressure greater than 65 mmHg [4]. It is recommended that if an additional vasopressor is needed, consider vasopressin [9].

Ventricular dysrhythmias

Ventricular dysrhythmias are the leading cause of mortality in the setting of TCA overdose. It is recommended that magnesium sulfate 2 g be administered IV followed by 3% hypertonic saline dosed at 1-3 mL/kg IV over 10 minutes [4]. Overdrive pacing for ectopy or bradycardia may be indicated.

Intravenous lipid emulsion therapy

Multiple case reports indicate the use of intravenous lipid emulsion therapy for large-volume TCA overdose [10,11]. It is believed that lipid emulsion therapy creates a lipid sink to bind TCAs. Successful outcomes have been reported. However, further studies are needed to clinically validate lipid emulsion therapy.

Intravenous glucagon therapy

Multiple case studies suggest that glucagon dosed at a 1 mg IV bolus may be effective for treating refractory hypotension or cardiogenic shock in the setting of TCA toxicity [12]. Glucagon is an endogenous, 29 amino acid, single-chain polypeptide secreted by the alpha cells of the pancreas [13]. It inhibits intestinal peristalsis and stimulates ketogenesis, glycogen lysis, gluconeogenesis, and lipolysis. Synthetic glucagon is believed to bypass the beta-adrenergic receptor and have a positive ionotropic and chronotropic effect directly on myocardial tissues [14]. It is not clinically recommended as further studies are needed to validate the utilization of intravenous glucagon in the setting of TCA toxicity.

Hemodialysis

Due to the large volume of distribution and highly protein-bound pharmacokinetics of TCAs, hemodialysis is not recommended.

Atypical antipsychotics

Antipsychotics are indicated in the treatment of psychological disorders including schizophrenia and bipolar disorder. They are utilized to improve mood, cognition, and behavior. Antipsychotics are organized into 1st, 2nd, and 3rd generations [15]. First-generation antipsychotics are typically linked to side effects such as weight gain, impaired glucose tolerance, and metabolic syndrome. Second-generation antipsychotics commonly referred to as atypical antipsychotics are thought to be as efficacious as 1st generation but differ in their side effect profile. They are used to treat schizophrenia and bipolar disorder. Ziprasidone is classified as a second-generation atypical antipsychotic with dopamine D2 and 5-HT2A receptor antagonism. It is also known to bind multiple serotonin receptors and block monoamine transport which prevents serotonin and norepinephrine uptake. It has a low binding affinity for muscarinic cholinergic M1, histamine H1, and alpha-1 adrenergic receptors. Ziprasidone is heavily metabolized in the liver via the primary productive pathway and CYP3A4 [16].

Toxicity

Common adverse reactions reported include QT/QTc prolongation, somnolence, neuroleptic malignant syndrome, orthostatic hypotension, metabolic syndrome, agranulocytosis, respiratory tract infections, extrapyramidal symptoms, dizziness, akathisia, abnormal vision, asthenia, vomiting, headache, and nausea [16].

Acute overdose

Most commonly atypical antipsychotics may present with an anticholinergic toxidrome including tachycardia, mydriasis, flushed skin, decreased sweat/salivary production, and acute urinary retention. Tachycardia, prolongation of the QT interval, and mild hypotension may manifest as a result of interactions with cardiovascular receptors. Neurologic manifestations may also include neuroleptic malignant syndrome and extrapyramidal symptoms [16].

TCA and atypical interactions

Clomipramine and ziprasidone co-administration are category Risk D with recommendations to choose alternative therapy (Table 3). Ziprasidone is thought to enhance the QT-prolonging properties of clomipramine. In the setting of acute ingestion and overdose, this places the patient at an increased risk for seizures and ventricular dysrhythmias which can lead to increased mortality.

**Table 3:** Second-generation atypical antipsychotics, mechanism(s) of action, and clinical effects.

Drug	Mechanism	Clinical Effect(s)
Clozapine	Dopamine antagonist, serotonin antagonist, partial serotonin agonist	Agranulocytosis, seizures, weight gain, myocarditis
Olanzapine	Dopamine antagonist, serotonin antagonist	Agitation, sedation
Quetiapine	Dopamine antagonist. serotonin antagonist	Tachycardia, orthostasis, sedation, delirium
Risperidone	Dopamine antagonist, serotonin antagonist	Dystonia, orthostasis
Ziprasidone	Dopamine antagonist, serotonin antagonist, partial serotonin agonist, serotonin & norepinephrine reuptake inhibitor	Akathisia, QTc prolongation

## Conclusions

Although TCAs and atypical antipsychotics are being prescribed in declining numbers, off-label uses persist. Those with relative contraindications including increased risk of suicidal behavior should not be prescribed these medications. Multiple treatment modalities exist with wavering evidence of support for TCA and atypical antipsychotic overdose. Overall, the mortality rate from TCA overdose without healthcare intervention is estimated to be 70%. When treated by emergency medicine physicians, the mortality rate decreased to approximately 3%. Through the clinical application of pharmacology, toxidromes, and expected clinical manifestations of side effects, emergency medicine physicians may be aptly prepared to provide life-saving interventions.
